# Annotations

**Published:** 1901-03-09

**Authors:** 


### ANNOTATIONS.

### Measles, Whooping Cough, and Education.

However enthusiastic one may feel in the cause of education, one cannot but look with a certain degree of suspicion upon that modern extension of the educational idea under the influence of which our elementary schools are to a large extent turned into *crèches* for the care of infants at an age when they certainly ought to be under their mothers' eyes. That these little "dots" between three and five years of age should be able to accompany the older children to school, where, if they can be kept awake, they are amused by some form of kindergarten entertainment until it is time for their sisters to go home again, is no doubt a very satisfactory arrangement from the point of view of the mother, whom it leaves free to work or gossip as may seem most suitable to her pocket or her inclination; but it is a very serious question whether the risk of disseminating infection which is caused by this accumulation of babies, at the most susceptible period of their lives, does not quite outweigh the very problematical advantages of the moderate amount of mechanical mental training given at school to children of this age—not a tithe of what they would receive from being chatted to by their mothers all the morning long, each infant with a whole mother to itself—instead of fifty babies in a row with one tired teacher to the lot.

Whooping cough and measles are the two most fatal maladies in that group of children's diseases, the spread of which is largely due to school attendance. During the ten years, 1889–1898, these two diseases caused nearly half of the whole zymotic mortality in London. Now, if we inquire of what age are the victims of these two fatal diseases, we find that 96 per cent. of all the deaths from whooping cough occur in children under five years old, while in regard to measles upwards of 90 per cent. are under five years old (Whitelegge). To come to exact figures we find, on referring to the last report of the Medical Officer of Health to the County of London, that Mr. Shirley Murphy gives the total deaths from measles in London during the year 1899 at 2,133, of which no less than 2,015 were in children under five years of age. Both in measles and whooping cough the majority of these deaths take place in the first and second year, but there can hardly be a doubt that these little ones become infected by those who go to school; and it is impossible to look at these figures (showing as they do that these diseases fall almost exclusively upon children under five years of age), not to mention those dealing with scarlet fever and diphtheria, to which we have already several times referred, without seeing how very largely the incidence of zymotic disease among children is favoured by the practice of sending very young children to school. Education, of course, we must have, and for the sake of getting education certain risks must be run and are doubtless justifiable. All that we wish to point out is this—that the educational advantages to these small children under five years of age are not great—are, indeed, very problematical—while it is to, and through, these babies that the danger comes. If they could be kept out of the schools until the age of five the period of greatest danger would then be over, and by so limiting the spread of infection there would not only be a great saving of life among these little children, but there would, probably, be a considerable diminution of that vexatious interruption to school life among the elder children which is constantly taking place from the introduction of infection into the schools.

### The Changing Style of Habitation.

In the Milroy Lectures delivered before the College of Physicians Dr. John F. Sykes, medical officer of health to St. Pancras, has undertaken a most interesting and important inquiry, his object being to ascertain how the health and vigour necessary for an active and useful life may best be maintained amid those new conditions of existence which are involved in that steady conversion of London into a city of flats which at the present time is so manifest on every side. He points out that the most serious health problem which now confronts us at the end of the Victorian reign, the most prosperous period of English history, was also the most serious problem met with towards the end of the Elizabethan reign, 300 years earlier, and the next most prosperous period of our history. At each period the cause and effect have been the same. Increased prosperity then as now acted as a stimulus to the growth of towns to such an extent that the crowding of the population into cubic space, and of houses upon square space, became a danger to the public health. Those were days when the relentless action of economic laws was hardly recognised, and when all things seemed possible to the law as laid down by our rulers. Hence most extraordinary Acts were passed, not to meet the demand for house-room, but to stop it. Among other things it was enacted that no new buildings, except for the better classes, were to be erected within three miles of London and Westminster, and no existing buildings were to be divided, and this apparently not being enough it was ordered by proclamation in 1602 that newly-built houses were to be pulled down. The problem then is no new one, but now it is recognised that the drift townwards is inevitable, that resistance to it is impossible even were it wise, and that what we have to do is to take care, if we can, that the crowded life, which we cannot prevent, shall be as little injurious as may be. A great change in the habits of the people is happening before our eyes. Not only is the growth and pressure of population in London causing overcrowding among the poor, but it is altering the mode of living among almost every class. The exclusiveness of the house and of the home is giving way before the steady extension of co-operative living. Among the affluent and middle classes mansions are now often shared by separate families; flats, with and without common commissariat arrangements, are springing up on every side; boarding-houses and residential clubs are increasing in number; and hotel life has become fashionable. Amongst the working classes existing houses are being more and more subdivided into tenements; flats of a small kind are being erected in increasing numbers; poor men's hotels or cubicle lodging-houses are introduced; and family lodging or boarding-houses are coming on the scene. Thus so far as the health of the people is concerned the great housing problem is not merely a question of where and by whom houses are to be erected, but is first and foremost a question of what sort of houses we are to aim at and what sort to allow; and as to this it is to be confessed that we know but little. Dr. Sykes has set himself a most interesting task.

### Gas Stoves.

We have received a long letter from Mr. Thomas Fletcher, F.C.S., the well-known maker of gas stoves, detailing a number of experiments made with the object of ascertaining what truth there is in the oft-repeated assertion that gas fires produce excessive "dryness" of air. The results of these experiments are such as to indicate, as indeed was only to be expected, that so far as hygrometric condition is concerned it is a matter of indifference whether a room is warmed by the heat radiating from red-hot cinders or red-hot asbestos lumps. Going further, Mr. Fletcher shows that the sense of dryness, which undoubtedly is to be felt in some rooms which are furnished with gas fires, is due, not to any real lack of moisture in the air, but to the presence of irritating products of combustion which ought to have gone up the chimney; in other words, is due to defective setting of the stove or defective draught in the chimney. Here, again, we think he is right. In many a stuffy gas-heated bedroom we have been able by aid of a bit of smouldering paper to show that the chimney was not doing its work, and that the products of combustion were coming into the room. If then we are, with Mr. Fletcher, to assume that when a gas-heated room feels "dry" it really does so because of the presence in the air of the always deleterious and sometimes poisonous products of combustion, we can readily understand how it is that gas fires cause headaches and choky throats. Mr. Fletcher appears to assume that it is the sulphur acids which do the harm. But in the products resulting from the combustion of gas in an ordinary incandescent gas stove there are many worse things than sulphurous acid, and until the plumbers learn to set gas stoves in such a manner that the chimney shall entirely remove all products of combustion from the room, or until the stove makers invent something which will so thoroughly consume the gas that nothing is left beyond water, carbon[illegible] sulphurous acid (the latter of which ought never [illegible] in large quantity), physicians will continue to look a[illegible] at gas as an ordinary means of continuous heating, u[illegible] as it undoubtedly is for temporary purposes.

### The Plague.

The somewhat rapid extension of the plague which has taken place during the past ten days in Capetown is a matter which cannot but be regarded with considerable anxiety. As was only natural, in view of the comparative propinquity of the South African ports to that great emporium of plague, Bombay, a port with which there has been constant communication, and in which plague has been constantly present since the war began, cases of plague have several times shown themselves at one place or another, but the disease has never seemed really to take root till recently. On this occasion, however, the outbreak has manifested all the well-known features of epidemic plague, including the occurrence of hidden cases. This we take it is one of the most serious indications of the gravity of any outbreak of plague. So long as rats are not affected one may always hope that it will be possible by simple measures to put a ring fence round the epidemic and prevent its spread; and so long as people will report the cases one knows exactly whom to isolate and what to do. But when the inhabitants hide their cases it is by no means an easy task to search them out. This has all along been the difficulty in Bombay, and it threatens to be so also in Capetown. From an epidemiological point of view nothing could be worse than the news that the corpses of unreported cases are being found, indicating as it does a complete lack of that discipline among the people, and that willingness to take part in preventive measures, which more than anything else tends to render such preventive measures effectual. No place could be more difficult to deal with than Capetown as it exists to-day, with its motley crew of mixed races, brought together accidentally from every quarter of the globe, entirely cut adrift from their normal surroundings, and most of them soaked in ignorance and superstition. The control of plague in such a place is sure to be a big business. Fortunately so far as our troops in the up-country are concerned several other ports are available, and Capetown, useful as it is as a base, is not absolutely essential.

### The University of London.

At the last meeting of the Senate of the University of London it was resolved: "That it be referred to the Faculty of Medicine to consider and report to the Senate as to the manner in which it may best carry out the duty stated in the following paragraph of Section LXXX. of the Statutes of the University: 'The Senate shall use its best endeavours, whenever practicable, to secure such common courses of instruction for internal medical students in the preliminary and intermediate portion of their studies under appointed or recognised teachers at one or more centres.'" This looks like business, for this is really the key to much that may happen under the new regulations. No amount of modification of the examination system will make a tithe of the difference to the relations between the University and the students that will be made at once by the University becoming itself responsible for the teaching, instead of merely examining up to a syllabus. It is to be hoped that the resolution will be taken into consideration at an early date, for if anything is to be fixed up in time for the October session there is no time to lose.

